# Supporting carers of stroke survivors to reduce carer burden: development of the Preparing is Caring intervention using Intervention Mapping

**DOI:** 10.1186/s12889-019-7615-2

**Published:** 2019-10-29

**Authors:** Jessica F. Hall, Thomas F. Crocker, David J. Clarke, Anne Forster

**Affiliations:** 1 Academic Unit of Elderly Care and Rehabilitation, Bradford Teaching Hospitals NHS Foundation Trust, Bradford, UK; 2 Academic Unit of Elderly Care and Rehabilitation, Leeds Institute of Health Sciences, University of Leeds, Leeds, UK

**Keywords:** Carer, Stroke, Burden, Intervention Mapping, Behaviour change, Intervention development, Carer needs, Qualitative, Systematic reviews

## Abstract

**Background:**

Burden is well documented among carers of stroke survivors, yet current evidence is insufficient to determine if any strategies reduce this negative outcome. Existing interventions for carers of stroke survivors typically involve supporting carers according to their individual needs through face-to-face interactions and provision of information including workbooks or educational guides. To date, no interventions have been developed using a method which systematically incorporates evidence, behaviour change theories, and stakeholder involvement to change the behaviours of carers and relevant individuals who support carers. This study aimed to develop a programme plan for a theory and evidence-based intervention to reduce burden in carers of stroke survivors.

**Methods:**

Informed by evidence from two systematic reviews and 33 qualitative interviews, the first four stages of Intervention Mapping were used to guide the intervention development process: 1) needs assessment; 2) identifying outcomes and objectives; 3) selecting theoretical methods and practical applications; and 4) creating a programme plan. Structured and facilitated involvement from stakeholders, including carers, researchers, and professionals from health and community services was integral to the intervention development process. Stakeholders helped to prioritise the focus of the intervention, develop the goals, outcomes and objectives for the programme, and generate and refine intervention ideas.

**Results:**

Stakeholders prioritised the need for carers to feel prepared before and during the transition from hospital to home as key to reducing burden. The proposed intervention ‘Preparing is Caring’ targets this need and involves providing and signposting carers to relevant information and support for practical and emotional needs. This is to be delivered before, during, and immediately after the stroke survivor’s transition from hospital to home by a person taking on a single point of contact role. It is comprised of multiple theory-based components including: training packages for information and support providers working with carers and wider staff teams, plus elements to support carers to feel prepared.

**Conclusions:**

We have developed a comprehensive programme plan for a multiple-component, theory and evidence informed behaviour change intervention aimed at preparing carers before and during the transition from hospital to home. Future work is required to refine, implement and evaluate the Preparing is Caring intervention.

## Background

Stroke remains a major illness, occurring more than 100,000 times a year in the UK [1] . Four out of 10 stroke survivors in the UK leave hospital requiring informal, non-professional care from relatives and friends [1]. These relatives and friends providing unpaid care are referred to as carers throughout this paper. Carers are most commonly female spouses [2] however, it is not uncommon for male spouses, parents and children to undertake the caring role [1].

The caring role typically involves assistance with daily activities, including physical care, and provision of emotional support [3, 4]. Providing care for stroke survivors is particularly demanding, due to the lack of preparation for managing the unexpected and often complex nature of the stroke sequelae [5, 6]. Consequences of caring include health, emotional and social difficulties [7, 8] as well as disrupted relationships, changes in roles, loss of autonomy and independence [9, 10]. There are also financial implications of caring, considering many carers struggle to remain in work [11]; the estimated loss of earnings per year per carer is over £11,000, amounting to an annual loss of £5.3 billion to the UK economy [12]. This is inefficient from an economic perspective and leaves carers at risk of experiencing poverty and exclusion [13].

Unsurprisingly, the burden of caring has become a significant health concern [14, 15]. Burden is a term commonly utilised in the healthcare literature since the early 1960s, however there is no single, agreed definition. As it takes in to account the multifaceted nature of burden and its associated factors, the following definition was adopted for this research: ‘the physical, psychological, emotional, social, and financial stresses that individuals experience due to providing care’ (George and Gwyther, 1986, pg. 253).

Studies have indicated 25–46% of carers experience substantial burden within the first 6 months of caring after stroke [16, 17]. Severity of burden experienced by carers in this early period is associated with numerous factors including carer and stroke survivor characteristics. Examples include greater stroke survivor disability; emotional difficulties among stroke survivors and carers, including feelings of stress, anxiety and depression; and increased time spent caring [15, 18]. Prolonged tiredness and deprivation of personal needs may lead to increased burden for some carers [19, 20]. Developing strategies that effectively address all these aspects of burden in carers of stroke survivors remains challenging.

A Cochrane review of non-pharmacological interventions for carers of stroke-survivors found insufficient evidence to conclude that the identified types of intervention (support and information, teaching procedural knowledge, psycho-educational) reduce negative outcomes such as carer burden [21]. The London Stroke Carer Training Course (LSCTC), a structured in-patient carer training programme, was identified as the intervention with the most potential [22]. However, a multicentre cluster randomised pragmatic trial of the LSCTC (*n* = 928) reported that this training programme did not reduce carer burden or increase patients’ functional independence [23]. A parallel process evaluation reported the training programme was difficult to deliver at this point in the stroke care pathway, as it competed with other priorities for stroke unit staff, and carers were experiencing stress related to their relative’s stroke [24]. A systematic review of trials published since the review by Legg et al. [21] highlighted that there is insufficient evidence to conclude that any existing intervention reduces negative outcomes in carers of stroke survivors [25].

Many existing carer support interventions involve supporting carers according to their individual needs through face to face interactions and provision of supplementary information including workbooks or educational guides. Examples include the Stroke Association Family support [26]; the Discharge Preparation Programme (DPP) [27] and the Timing it Right Stroke Family Support Programme (TIRSFSP) [28]. However, in addition to finding no clear evidence of effectiveness, the review highlighted that many interventions have been developed without theory and evidence based understanding of a problem before formulating a solution. Where theory has been incorporated, descriptions of how theoretical components relate to intervention components are often inadequate [25].

Furthermore, no existing interventions have been developed using an approach which guides the selection of existing theory and evidence to focus on changing the behaviour of professionals who support carers to reduce negative outcomes in carers of stroke survivors in the transition from hospital to home. As we report later, it is likely that the behaviours of carers and professionals need to change to reduce negative outcomes among carers.

Using an Intervention Mapping approach [29] to develop a multi-component intervention, incorporating theoretical methods and practical applications to target the behaviours of both carers and professionals could potentially reduce and manage carer burden. In this paper we describe how this approach was applied as part of a doctoral study to develop a detailed programme plan for an intervention designed to reduce burden by ensuring carers of stroke survivors are more prepared before and during the transition from hospital to home.

## Methods

Intervention Mapping [29] is a six stage theory and evidence informed approach for developing behaviour change interventions which is based on three overarching perspectives (1. socio-ecological approach; 2. multi-theory and evidence based approach; 3. stakeholder participation). Intervention Mapping uses many technical terms. Table 1 has been included to provide clarity on the content that follows.

**Table 1:** Key Intervention Mapping terms

Key term	Description
Behavioural outcomes	Expected outcomes of the intervention that are specified in terms of overall behaviours to be performed by the individual
Environmental outcomes	Expected outcomes of the intervention that are specified at the levels beyond the individual e.g. interpersonal, organisational, community
Performance objectives	An expanded list of the specific behaviours and actions that when performed together produce the behavioural and environmental outcomes
Theoretical determinants	Constructs from theories that influence whether individual behaviours and behaviours of environmental agents can be changed; examples include knowledge, skills and beliefs about capabilities
Change objectives	The change objectives state what the intervention should modify to influence performance objectives to achieve the behavioural and environmental outcomes
Matrices of change	The matrices of change objectives outline the most immediate change to be addressed by the intervention and provide a basis for selecting theoretical methods and practical applications for the intervention in the next stage of Intervention Mapping
Theoretical methods	The theoretical methods are general techniques for influencing the theoretical determinants of behaviours and environmental conditions. Examples include modelling and belief selection.
Practical applications	Practical applications are the means by which the theoretical methods are delivered in a way that fit the relevant population and content. An example is video clips including role play

In this study, stages one to four of Intervention Mapping [29]^1^ (Table 2) were used to develop the intervention. Stages five and six (implementation and evaluation) will be incorporated in a future research project.

**Table 2:** Intervention Mapping stages 1–4 based on Bartholomew et al. [19]

1) Needs assessment (Logic model of the problem)	• Understanding the problem and the factors that influence the problem to create a logic model of the problem. • Establishing the overall programme goal.
2) Developing programme outcomes and performance objectives (Logic model of change)	• Considering what needs to be achieved to reach the overall programme goal and developing a logic model of change. This involves: ○ Stating the behavioural and environmental outcomes of the intervention. ○ Specifying the performance objectives to reach the outcomes ○ Selecting appropriate theoretical determinants for the matrices of change ○ Developing matrices of change
3) Selecting theoretical methods and practical applications (Programme design)	• Focusing on how the goals, outcomes and objectives can be achieved by: ○ Generating programme ideas. ○ Selecting theoretical methods ○ Selecting appropriate practical applications
4) Creating an organised programme plan (Programme production)	• Drawing ideas together to create detailed plans for a coherent intervention including a programme, scope and sequence document and design documents.

Intervention Mapping was chosen because it is a well-established framework for developing interventions which fulfils Medical Research Council (MRC) recommendations for developing complex interventions [31]. It provides structure for integrating theories and evidence and encourages clear documentation of the causal assumptions on which the intervention is based. Underpinned by the socioecological model [32], the approach also acknowledges that human behaviours and interventions to address behaviours are part of a complex system that can be influenced by factors at multiple levels [33]. Use of Intervention Mapping ensures the behaviours of individuals beyond carers such as family, friends, professionals, and service providers are considered in the management of carer burden. These factors, together with involvement from stakeholders provided the potential to address the complexities attached to caring, and helped to ensure that an intervention to reduce burden is grounded in the experiences and needs of carers.

### Stakeholder involvement

In this study, a stakeholder group was formed as an integral part of the intervention development process. Six carers were recruited in total. Five carers were approached at a local carers group; four agreed to participate, and one declined due to ill health. Twenty invitations were sent to potential carers via stroke survivors through a research register stored at the Academic Unit of Elderly Care and Rehabilitation (AUECR), Bradford Teaching Hospitals NHS Foundation Trust. Two carers identified from this register agreed to participate. A range of professionals from health and social care services and researchers were also approached and invited to participate. Three health professionals working in NHS settings, and three researchers working at the AUECR were known to the research team. They were therefore approached based on their knowledge and experience in supporting carers in the acute phase of care. Two professionals from carers’ support services were selected and approached via their organisations to gain a differing perspective on supporting carers at differing phases in the care trajectory. Table 3 provides further details about their roles and relevant experience.

**Table 3:** Stakeholders included in the Intervention Mapping process

Stakeholders	
• Six carers, all female and providing care for between 1 and 10 years (four attended all groups, two withdrew due to personal circumstances after two groups)	
• A PhD student and two senior researchers with experiences of developing interventions for stroke survivors or carers using behaviour change frameworks and models including Intervention Mapping and the Behaviour Change Wheel	
• Three National Health Service health professionals including a therapy co-ordinator, physiotherapist and a stroke nurse specialist	
• Two professionals from third sector carer support services (carer support and secondary care worker, information specialist)	

**Fig. 1:**
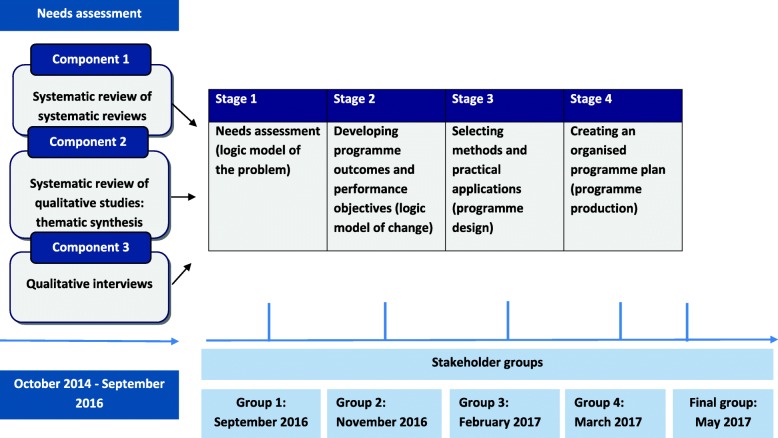
An overview of how stakeholders were involved in the Intervention Mapping process

Figure 1 provides an overview of the process and timelines, including when stakeholders were involved in the Intervention Mapping process, and the three components of work that contributed to the needs assessment (stage 1). These components outlined in the sections that follow.

The lead author convened and led five stakeholder meetings over a 9 month period. In all meetings, stakeholders were split into smaller groups to complete tasks and provided feedback to the wider group. Interactions in small groups and feedback were audio-recorded and documented on worksheets specifically designed for each task. In meetings with the stakeholders, use of technical terminology associated with Intervention Mapping such as determinants, matrices, and performance objectives was avoided. However, using carefully designed stakeholder materials meant stakeholders contributed to key decisions and their ideas could be integrated into the development work carried out away from the groups. Development work was facilitated by ad-hoc meetings aside from the groups with other researchers with expertise in developing interventions using behaviour change approaches.

The following sections provide a summary of how the first four stages of the Intervention Mapping process were applied to the problem of carer burden, resulting in a detailed programme plan for the intervention.

### Stage 1: needs assessment: logic model of the problem

The process began with an in-depth needs assessment to understand more about the problem, carer burden. This involved two key steps:

- Gathering the evidence and developing the logic model of the problem (referred to in this study as the logic model of burden)
- Establishing intervention priorities and developing the programme goal

Both are discussed in more detail in the sub-sections that follow.

#### Gathering the evidence and developing the logic model of burden

**Fig. 2:**
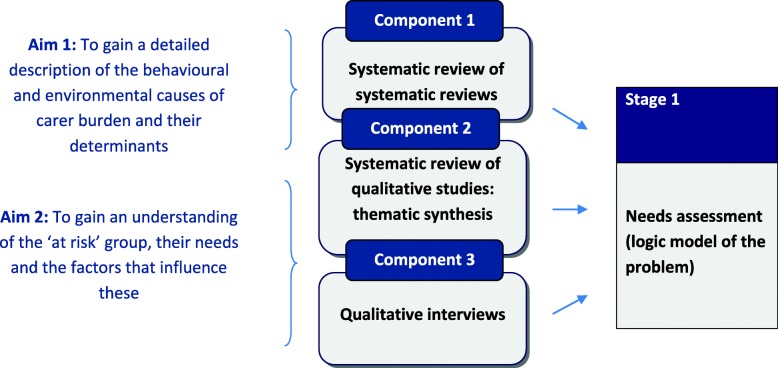
Aims and components of work contributing to the needs assessment

Evidence for informing a logic model of burden was gathered in three components of work contributing to the needs assessment that sought to address two main aims [29] outlined in Fig. 2. The components are summarised in this section with attention to how these informed the logic model of burden (Additional file 1 with supporting key in Additional file 2).

#### Component 1: systematic review of systematic reviews

A systematic review of reviews was conducted using a comprehensive search strategy in seven databases between 2010 and 2015, to establish the factors that influence burden in carers of stroke survivors and other longer- term conditions. More specifically, this sought to identify the behavioural and environmental conditions associated with carer burden and their determinants, together with carer and patient characteristics that influence carer burden. These factors were organised into the logic model of burden.

This work informed the decision to review existing evidence and conduct further qualitative research which addressed the second needs assessment aim and continued to inform the logic model of burden; adding to the behavioural and environmental factors and their determinants, necessary for intervention development.

#### Component 2: systematic review of qualitative studies: thematic synthesis

The second component was an update of a systematic review [5]. A comprehensive search strategy was used to identify studies from eight databases between 2005 and 2015. The review sought to establish carers’ needs, how they change over time, and the barriers and facilitators to addressing needs. Barriers and facilitators were identified in accordance with different levels of the socio-ecological model [32].

Findings from the thematic synthesis built upon the evidence presented in the systematic review of systematic reviews (component 1), added to the logic model of burden and provided a more in-depth understanding of the caring experience in carers of stroke survivors. More specifically, the identified needs provided a contextual understanding of experience and a potential basis for prioritising the intervention focus in later stages of Intervention Mapping. In the third component there was more focus on how needs and the barriers and facilitators to addressing needs changed over time.

#### Component 3: qualitative semi-structured interview study

A thematic analysis of 33 semi-structured interviews in two groups of carers was conducted by the first author to extend the review evidence by adding to understandings about how needs, barriers and facilitators change over time. In group one, carers were recruited in inpatient stroke units (acute and rehabilitation) in a local teaching hospitals trust; they took part in three semi-structured interviews. The first interviews took place within the first 4 weeks after the stroke survivors’ discharge from hospital (topic guide available in Additional file 3), the second and third were each separated by 3 months (topic guide available in Additional file 4). In group two, carers who had been providing care between nine and 36 months were recruited via an established research register and a local carers group. They took part in a single interview (Additional file 3). In both groups of carers, written consent was obtained prior to conducting the interviews.

Behavioural and environmental barriers identified in this empirical study informed the overall logic model of burden. The facilitators provided more potential solutions to be considered later in the intervention development. The logic model of burden presents a range of behavioural and environmental factors, and their determinants that could lead to carer burden, related to numerous and varied carer needs (Additional file 1).

#### Establishing intervention priorities and developing the programme goal

The next step involved establishing intervention priorities. Bartholomew et al. [29] specified that this should be achieved by identifying groups at greater risk of the health problem, or considering the magnitude between ‘what is and what could be’ ( [19], p., 226). This and more recent guidance [30] is abstract and provides little indication for how to do this, particularly with involvement from stakeholders.

The intervention focus was prioritised through a series of tasks involving stakeholders. The first task was designed to elicit stakeholders’ thoughts on carer burden. Stakeholders were presented with a mind-map of factors that contribute to burden in carers of stroke survivors and other longer-term conditions, based on the systematic review of systematic reviews evidence (component 1). They were asked to provide feedback to establish whether these findings accorded with their own experiences.

Stakeholders were then asked to prioritise carer needs based on their importance for reducing carer burden. They were presented with 11 cards, each including a different ‘carer need’ based on evidence from the thematic synthesis and qualitative interviews (components 2 and 3). Through discussions in small groups and as a whole, the stakeholders prioritised one need: ‘Carers need to feel prepared before, and during, the transition from hospital to home’.

To re-build the logic model of burden so that it was more specific to the prioritised need, rather than burden as a whole, it was important to understand more about this unaddressed need from a problem perspective. This was achieved through providing stakeholders with two further tasks. Firstly, stakeholders were asked to discuss specific examples of being or feeling unprepared before and during the transition from hospital to home. Secondly they discussed and wrote down the factors that resulted in being and feeling unprepared before and during the transition from hospital to home. The outputs of these tasks informed the logic model of the prioritised problem and the overall programme goal: ‘Ensuring carers feel and are prepared, before, during, and following the transition from hospital to home*’* (Additional file 5 with supporting key in Additional file 2).

### Stage 2: developing programme outcomes and performance objectives (logic model of change)

At this stage, the focus shifted from problems to the change process. The logic model of change (Additional file 6) is the output of this stage; this outlines pathways of the intended programme effects rather than pathways to identify causes of the problem [34]. Four steps were included in developing the logic model of change. Firstly, behavioural and environmental outcomes were established; secondly, performance objectives were created for each outcome; thirdly, appropriate theoretical determinants were selected for the matrices of change; lastly, the matrices of change were developed which included numerous change objectives (refer to Table 1 for definitions of these key terms).

#### Establishing behavioural and environmental outcomes

The programme goal and the logic model of the prioritised problem guided the development of behavioural and environmental outcomes that were informed by relevant levels of the socioecological model [32] e.g. individual, interpersonal, organisational.

#### Developing performance objectives

Performance objectives were generated based input from stakeholders and other evidence. Stakeholders were asked to record on a worksheet what different individuals and services (e.g. carers, professionals, family, friends, and peers) could do to reach the programme goal and outcomes. The worksheet provided a framework of responses informed by the socio-ecological model [32]. Other evidence included:

- Behavioural and environmental factors included in the logic model of the prioritised problem
- Facilitators that were identified in components two and three and previous similar work focused on developing longer-term support stroke survivors and their carers (LoTS2Care) [35]
- Theories of information seeking [36, 37] identified through rapid scoping of the literature.

The performance objectives contributed to the matrices of change created during this stage.

#### Selecting theoretical determinants

To create the matrices of change objectives, theoretical determinants from the logic model of the problem (in this case the prioritised problem) are mapped against the performance objectives. The determinants in the logic model of the prioritised problem were largely a-theoretical. They were either based on discussions with stakeholders, or findings from studies where theories were not applied to understand carers’ experiences. They did not map directly onto a single, unifying behaviour change theory but many determinants mapped on to the 14 domains in the Theoretical Domains Framework (TDF) [38], hence this framework was chosen.

#### Developing matrices of change objectives

Matrices of change objectives were created for each behavioural and environmental outcome. Each change objective states the most immediate change to be made by the intervention to achieve performance objectives and behavioural and environmental outcomes. They provide a basis for selecting theoretical methods and practical applications for the intervention in the next stage of Intervention Mapping.

#### Developing the logic model of change

The results of this stage were captured in the logic model of change (Additional file 6) which includes the programme goal, behavioural and environmental outcomes, performance objectives, determinants of behaviours, and change objectives.

### Stage 3: selecting theoretical methods and practical applications (programme design)

Stage three of the Intervention Mapping process involves two key steps: Generating programme ideas, then selecting theoretical methods and appropriate practical applications.

**Fig. 3:**
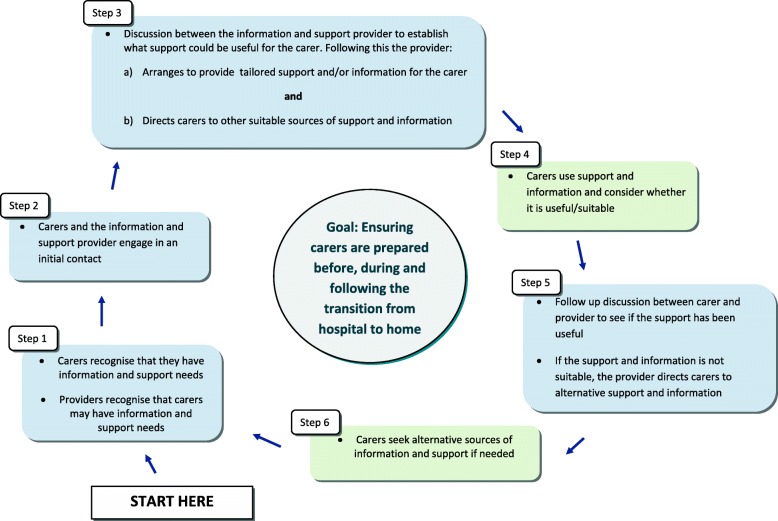
Flow diagram based on consolidated performance objectives which outlines the behaviours carried out by different individuals (carers and professionals) to achieve the programme goal

Stakeholders helped to generate programme ideas through their involvement in two tasks. Prior to engaging in the tasks, stakeholders were presented with a flow diagram (Fig. 3) to update them on the project progress in a manageable, understandable way since their last contributions to the performance objectives. The diagram included six steps based on consolidated performance objectives, which outlined the behaviours that must be carried out by different individuals (carers and professionals) to achieve the overall goal.

In the first task stakeholders were asked a series of questions related to the contextual factors of the behaviour change intervention (e.g. where, how, who, what and when). In the next task, stakeholders were asked to note ideas on another worksheet about the design and content, delivery preferences, and resources and materials. These tasks provided a structured approach to generating intervention ideas without constraining their creativity. The theoretical methods underpinning the required change i.e. the ‘active ingredients’ were yet to be established, hence the importance of the next steps.

For each programme idea, theoretical methods and appropriate practical applications to address theoretical methods needed to be selected. To aid the selection process, Bartholomew et al. [29] outlined numerous tables of theoretical methods linked to commonly used theoretical determinants. In this study, adaptations were made to the process to streamline the selection of theoretical methods. Theoretical determinants from the TDF [38] were grouped according to the theoretical methods used to target them (see columns 1 and 2 in Table 4). Then a further condensed grouping of determinants was created in a language that would be appropriate for discussion with stakeholders (column 3).

**Table 4:** Theoretical determinants and theoretical methods

Determinants from the TDF [38]	Grouped determinants according to the similarities in theoretical methods used to target them [29]	Translation into language appropriate for stakeholders
• Knowledge	• Basic methods at the individual level • Methods to increase knowledge	• Knowledge
• Skills • Memory, Attention, and Decision Processes • Beliefs about Capabilities	• Basic methods at the individual level • Methods to change skills, capability, and self- efficacy to overcome barriers	• Skills and Decision-Making Abilities
		• Confidence in their own capabilities
• Social/ Professional Role and Identity • Social Influences	• Basic methods at the individual level • Methods to change social influence	• How they see their role/professional role
• Beliefs about consequences • Optimism	• Basic methods at the individual level • Methods to change attitudes, beliefs, and outcome expectations	• Beliefs and attitudes
• Reinforcement	• Basic methods at the individual level	• Reasons or incentives
• Intentions • Goals • Behavioural Regulation	• Basic methods at the individual level • Methods to change habitual, automatic, and impulsive behaviours	• Intentions and goals
• Emotion	• Basic methods at the individual level	• Emotions
• Environmental context and Resources	• Basic methods at the individual level	• Having the Right Context for the Intervention and Resources in Place

Stakeholders were presented with cards outlining the determinants in column 3 and discussed which could potentially be targeted in the intervention. Their feedback in addition to evidence from theories and the logic model of the prioritised problem was considered when choosing which domains of the TDF would be selected to map the theoretical methods and practical applications.

To document the theoretical methods and practical applications, a series of seven tables (one for each group of determinants) were created. Based on relevant guidance [39], each table included columns for the determinants, change objectives, theoretical methods, parameters, practical applications and how population, context and parameters were considered throughout all these decisions.

This part of the process was adapted to make it more manageable. Instead of listing every change objective from the matrices of change, these were consolidated using the flow diagram as a structure for these tables (Fig. 3). In each table, change objectives were listed under the relevant step in the flow diagram (based on consolidated performance objectives) and theoretical methods were matched to each change objective. By considering the parameters, the conditions under which methods are shown to be effective were kept in mind during the translation from methods to applications through to overall programme ideas.

### Stage 4: creating an organised programme plan (programme production)

Having selected the theoretical methods and practical applications, and gained feedback from stakeholders a programme plan of the intervention was produced. This included a document outlining the scope and sequence of the intervention, and design documents which provided a detailed outline of the proposed intervention components.

## Results

This section focuses on outputs from stages one to four of the Intervention Mapping process that were described within the methods section.

### Stage 1: needs assessment: logic model of the problem

#### Evidence and the logic model of burden

This section provides the results from the three components of work that informed the logic model of burden and shaped the overall programme goal.

#### Component 1: systematic review of systematic reviews

Fourteen systematic reviews (*n* = 612 studies, some of which were included in more than one review) were identified from seven databases and included in the review. A range of behavioural and environmental conditions and their determinants, and patient and carer characteristics were identified. Findings suggested that interventions may need to target both behavioural and environmental factors, because carers’ experiences are influenced by their own behaviours and the external environment, including the behaviours of others responsible for the provision of support.

The determinants of behavioural and environmental factors also had implications for intervention development. Carers’ perceptions and degree of satisfaction with support influence carer burden, suggesting the importance of ensuring support meets their needs. Evidence for different coping types that reduce burden was mixed and based on carers of people with dementia. However, this suggested the possibility of carers protecting themselves from experiencing burden by developing coping strategies, if encouraged with the appropriate support.

All of the identified behavioural and environmental conditions associated with carer burden, and their determinants, together with carer and patient characteristics that influence carer burden were organised into the logic model of burden (Additional file 1).

#### Component 2: systematic review of qualitative studies: thematic synthesis

Forty eight studies published since the original review were identified from eight databases and included in a thematic synthesis where nine descriptive themes and nine analytical themes were identified. Findings indicated that carers have varied needs relating to different aspects of care, confirming experiences and needs reported in previous reviews [5, 40, 41].

The review outlined barriers and facilitators at different levels of the socio-ecological model [32] that were instrumental in how carers addressed different needs. The review findings suggested a tailored intervention, targeting multiple levels of the socio-ecological model could be appropriate for addressing carers’ needs; in addition to gaining a balance between promoting a proactive carer and ensuring that appropriate support and information is available.

The identified needs provided a contextual understanding of experience and a basis for prioritising the intervention focus in later stages of Intervention Mapping. Behavioural and environmental barriers e.g. avoiding asking professionals for support, and reduced social networks over time informed the developing logic model. Facilitators helped to provide a more complete picture of experience in addition to barriers. They also outlined potential solutions to be considered later in the intervention development e.g. assisting carers to develop support networks. It was however difficult to determine exact time periods when needs emerged or how these and the barriers and facilitators to addressing needs changed with time. This was explored in the third component.

#### Component 3: qualitative semi-structured interview study

Twenty carers took part in 33 interviews across the two groups. In group one the number of identified needs differed with time (time 1 = 9, time 2 = 8, and time 3 = 6). In group two, nine needs were identified, all of which related to different aspects of caring. Findings from group one provided a nuanced understanding of how needs change over time in the same group of carers, highlighting when some needs should be addressed. Group two findings provided insight into how needs and the barriers and facilitators to addressing needs compare in the later care experience.

Behavioural and environmental barriers identified in this empirical study informed the overall logic model of burden. Examples included ‘providing practical care without preparation’, and ‘professionals do not always provide initial guidance to reassure carers.’ The facilitators provided more potential solutions to be considered later in the intervention development such as encouraging carers to actively seek support. Additional file 1 includes the full logic model of burden, Table 5 summarises some of the key behavioural and environmental factors and determinants that were either specific to stroke in the review of reviews (stroke in brackets in Additional file 1 and in the table) or were evident in both the thematic synthesis and qualitative interviews (bold and italic in Additional file 1 and in the table).

**Table 5:** Summary of key behavioural and environmental factors and determinants that influence carer burden

Behavioural factors	• *Avoiding asking for support from family and friends* • *Engaging in care role/ duties restricts time to self* • *Focusing on the stroke survivor means little time for engaging in own activities/time to self*
Personal determinants (carers)	• Build-up of strain over time (stroke) • *Reluctance to ask family and friends for support due to feeling bothersome, not wanting to burden them* • *Lack of knowledge around who to ask and where to access support and information* • *Difficulties thinking about the future/worries about the future* • *Fears of stroke survivor safety, falling and setbacks, reoccurrence of stroke*
Environmental factors	• Rehabilitation support that lacks continuity at home and in the community (stroke) • Services and organisations that fail to engage in adequate planning as part of the stroke survivors’ rehabilitation (stroke) • *Lack of support provided by professionals initially, influences coping with changed relationships, coping and managing practically* • *Lack of information provided about cause of stroke, extent of recovery, expectations before and during the transition from hospital to home, access to support, available support (before and following the return to home)*
Personal determinants (professionals, family, friends, peers)	• *Professionals lack time to professionally prepare carers for the transition from hospital to home* • *Friends and family struggle to understand the situation*

#### Intervention priorities and the programme goal

In the first task presented to stakeholders which was designed to elicit stakeholders’ thoughts on carer burden, they largely agreed with the factors presented in the mind-map. They also provided additional insights into these factors in a stroke specific context. In the second task which involved prioritising 11 needs, through discussions in small groups and as a whole, the stakeholders prioritised one need: ‘Carers need to feel prepared before, and during, the transition from hospital to home’.

In a further task, stakeholders discussed examples of being prepared before and during the transition from hospital. Key findings included practical and emotional struggles, feeling uncertain, and unaware that things would be different. Carers emphasised feeling emotionally unprepared, which they found harder to manage than the physical aspects of care. Where carers faced practical struggles, these included: managing medications, and physically getting the stroke survivor up the stairs or moving in and out of bed. Uncertainty around expectations was attached to changes in the stroke survivors and resultant impacts on relationships and roles around the home. Without adequate preparation carers often returned home without the realisation that their lives, sense of normality and independence would be different.

Stakeholders then discussed and wrote down the factors that resulted in being and feeling unprepared before and during the transition from hospital to home. Factors broadly related to information and support e.g. lack of follow up support from professionals, not getting enough information unless signposted to charities, reluctance to approach staff for information, and not being in the right frame of mind to take in information. These findings were important for establishing the nature of an intervention, for example, whether it would involve both emotional and practical preparation.

Discussions with stakeholders during these tasks also indicated that for carers to feel prepared may require intervention beyond the initial transition to home, to the time shortly following this. During this time, carers reported they were still engaging in practical preparation and realised that they were still emotionally unprepared. This was taken into account in the development of the overall programme goal: ‘Ensuring carers feel and are prepared, before, during, and following the transition from hospital to home*;’* and the logic model of the prioritised problem (Additional file 5).

### Stage 2: programme outcomes and performance objectives (logic model of change)

#### Behavioural and environmental outcomes

The programme goal and the logic model of the prioritised problem guided the development of 15 behavioural and environmental outcomes that were informed by relevant levels of the socio-ecological model [32] e.g. individual, interpersonal, organisational (Table 6).

**Table 6:** Behavioural and environmental outcomes

Behavioural outcomes	
Individual (carer):	
• Carer gains information while the stroke survivor is in hospital • Carer gains support while the stroke survivor is in hospital • Carer gains information following the transition from hospital to home • Carer gains support following the transition from hospital to home	
Environmental outcomes:	
Interpersonal (Professionals, family, friends, and peers)	
Professionals:	
• Professionals provide useful information to carers while the stroke survivor is in hospital • Professionals provide useful support to carers while the stroke survivor is in hospital • Professionals provide useful information to carers following the transition from hospital to home • Professionals provide useful support to carers following the transition from hospital to home	
Family, friends, and peers:	
• Family, friends, and peers provide useful information to carers while the stroke survivor is in hospital • Family, friends, and peers provide useful support to carers while the stroke survivor is in hospital • Family friends and peers provide useful information to carers following the transition from hospital to home • Family friends and peers provide useful support to carers following the transition from hospital to home	
Organisational (Service)	
• Services including (hospitals, carer charities, and support groups) promote the involvement of carers to ensure that they are provided with the required information and support during the time when the stroke survivor is in hospital. • Services including (hospitals, carer charities and support groups) promote the involvement of carers to ensure that they are provided with the required information and support following the transition from hospital to home	
Community (relationships among organisations)	
• Services work together to ensure a continuity of support and information for carers before, during and following the transition from hospital to home	

Contributions from stakeholders and findings from the needs assessment indicated that ensuring carers gain support and information is important for their preparation. This is reflected in the behavioural and environmental outcomes.

#### Performance objectives

Stakeholders were influential in developing 168 performance objectives across the 15 behavioural and environmental outcomes.

#### Theoretical determinants

The determinants in the logic model of the prioritised problem mapped onto nine of the 14 domains of the TDF [38] hence, this was chosen. The matrices of change were developed with all 14 domains to ensure that the five domains that did not map onto the evidence in the logic model of the prioritised problem were not discarded prematurely. The five domains included reinforcement, intentions, goals, behavioural regulation, memory, attention, and decision-making processes.

#### Matrices of change objectives

Fifteen matrices of change were created (one for each behavioural and environmental outcome). These provided a basis for selecting theoretical methods and practical applications in the next stage of the process.

#### Logic model of change

The results of this stage of Intervention Mapping are presented in the logic model of change (Additional file 6).

### Stage 3: methods and practical applications (Programme design)

As part of generating the programme ideas, stakeholders answered a series of questions to clarify contextual factors of the behaviour change intervention (e.g. where, how, who, what and when). Stakeholders agreed when, how and where carers would benefit from help to gain the support and information required to feel prepared. They suggested this should be during the carers’ time in hospital, continuing across the transition from hospital to home, in face-to-face discussions at hospital, then either in the carers’ own homes or GP surgeries. It was less clear who should take on the role of the information and support provider. Stakeholders suggested someone in the stroke ward, community-based staff, GPs, or an additional role. Stakeholders’.

ideas about ‘what’ carers could be given during discussions with the information and support provider to facilitate preparation included: a log of contacts on either a key ring or credit-card-sized card; access to a helpline; an ‘in case of’ plan to guide carers through scenarios (e.g. if I struggle, I will do x, y, and z).

In the next task, stakeholders noted ideas on another worksheet about the design and content, delivery preferences, and resources and materials. Preferences for the programme design and content were: a training package including modules with interactive and written content for ‘information and support providers’ and a session for staff in the wider teams. Delivery preferences included face to face training and supervision sessions where staff members meet competencies. Ideas about resources and materials included appropriate funding, training package materials and resources for materials provided to carers e.G. *key* rings.

Using the cards provided, stakeholders discussed which determinants could potentially be targeted in the intervention. They focused on determinants related to the information and support providers, these included: beliefs about capabilities; social and professional role identity; social influences; emotion; knowledge; skills; memory, attention and decision processes; and environmental context and resources.

As part of the process where theoretical methods and practical applications are selected, stakeholders engaged in a task which involved selecting determinants that could potentially be targeted in the intervention. They focused on determinants related to the information and support providers, these included: beliefs about capabilities; social and professional role identity; social influences; emotion; knowledge; skills; memory, attention and decision processes; and environmental context and resources.

Considering the feedback from stakeholders, as well as evidence from theories and the logic model of the prioritised problem, all 14 determinants of the TDF [38] seemed relevant to some extent. A lack of theory-based evidence for these behaviours compared to other typical health behaviours such as physical activity meant it was difficult to prioritise some over others. Therefore, all determinants were considered when the theoretical methods and practical applications were selected.

As described in the methods section, a series of tables were produced to document the selection of theoretical methods and practical applications.

### Stage 4: organised programme plan (Programme production)

Following the process outlined in the methods, a final plan including the scope and sequence of the intervention and a series of design documents were produced. This section provides an overview of the proposed intervention named ‘Preparing is Caring’ and a summary of what is included in the documents.

Preparing is Caring is comprised of various intervention components incorporating theoretical methods. These include: A training package for information and support providers working with carers (including an induction plus five key modules and on-going supervision sessions); an additional training session for the wider staff team. Elements to support carers to feel prepared include brief written information to introduce the information and support provider; ongoing face to face discussions; an ‘in case of’ plan for carers, including relevant contact numbers for practical and emotional needs; key rings or cards of key contacts; and access to a helpline.

The training package for the information and support provider is to equip them to support carers to feel prepared before, during and following the transition from hospital to home. This targets multiple determinants including skills, knowledge, and beliefs about capabilities across five modules. The module content is based on multiple different theoretical methods (e.g. modelling, persuasive communication) and practical applications (e.g. role plays, discussions following video clips). This can be made available for face-to-face delivery and/ or as an online resource.

The supervision sessions focus on meeting competencies. This idea was favoured by the stakeholders as an opportunity for ongoing learning and to provide evidence for the impacts of working with carers. These sessions aim to target multiple determinants including beliefs about capabilities and skills. The additional training for the wider staff team is intended to promote a culture of supporting carers in addition to stroke survivors.

The information and support providers will provide carers with additional elements to assist preparation before, during and following the transition from hospital to home. As with the training package, each element is based on varied theoretical methods that target numerous determinants including knowledge, beliefs about consequences, and reinforcement.

Stakeholders contributed to intervention ideas by expressing their preferences and awareness of constraints regarding the intervention context, delivery (who and how), time spent training, content of modules and how this could be implemented within services. Their preferences informed the programme, scope, and sequence document, created for the training package elements of the intervention. This outlines details of the five key modules, key messages, and activities. Their preferences also informed the design documents that are traditionally created for each element of the intervention. The design document for the training package includes module titles, activities, goals, and design features (including interpersonal features e.g. conversations, teaching approaches). The design document for the elements to support carers to feel prepared includes a description for each element, detailed outline of content and their impact.

## Discussion

This paper describes the process of using Intervention Mapping to develop a programme plan for the ‘Preparing is Caring’ intervention. To our knowledge, ‘Preparing is Caring’ is the first proposed complex intervention aimed at reducing burden in carers of stroke survivors that has been developed using Intervention Mapping. The Intervention Mapping process, incorporating evidence from empirical research, review data, behaviour change theories and collaboration with a stakeholder group is consistent with MRC framework guidance [31] and has the potential to provide a valuable contribution to the development of interventions for carers of stroke survivors.. This contribution is important, given that current evidence from randomised controlled trials is insufficient for determining which interventions are most effective for reducing burden in carers of stroke survivors.

The ‘needs assessment’ stage provided more clarity on how needs were determined compared to existing interventions aimed at carers of stroke survivors where it is unclear what evidence was used to determine carers’ needs or how intervention materials sought to address needs [26, 27, 42–44]. Involving stakeholders in prioritising the focus of the intervention meant that intervention materials were designed with a more specific purpose and focus to address one key need.

Preparing is Caring differs from all existing interventions for carers of stroke survivors due to the focus on changing how professionals support carers in the transition from hospital to home. This can be traced to the use of Intervention Mapping which acknowledges that behaviours and interactions are part of a complex system, influenced by multiple levels [33]. Other similar interventions have provided opportunities to interact with professionals (e.g. Stroke Association Family support [26]; Discharge Preparation Programme (DPP) [27] and Timing it Right Stroke Family Support Programme (TIRSFSP) [28]), rather than seeking to change the way professionals support carers. The multiple components included in Preparing is Caring also differ from materials provided in existing interventions because they are based on theoretical methods and practical applications that target both information and support provider and carer behaviours.

### Strengths and limitations

The emphasis on working from a problem towards a solution avoided skipping to solutions that were based on little evidence. Furthermore, using the socio-ecological model to frame outcomes, objectives and matrices, highlighted the importance of identifying opportunities for behaviour change beyond the individual i.e. carers of stroke survivors. This provided a more complete picture of the changes required to address the complex problem of carer burden, highlighting that interventions should consider the nature of interactions between professionals and carers and acknowledge wider environmental influences on behaviours.

It is possible that a different group of stakeholders (e.g. carers with more varied characteristics and other professionals e.g. clinical psychologists, stroke physicians, and occupational psychologists) would have a different influence on the intervention that has been produced. However, the intervention development process was not solely based on input from stakeholders. It was also based on a large body of evidence contributing to the needs assessment, gathered using rigorous methods. Collaboration with this stakeholder group in fact exemplified the importance of developing an intervention that is grounded in how they understand and experience burden. This was an important contribution, given that a previous qualitative review [5] and qualitative evidence from the needs assessment indicated that carers rarely use the term ‘burden’ in their narratives about their care experiences. The management of stakeholder involvement throughout the process is also a strength of this research. This also provides an important example given that there is little published guidance on how to involve them in decisions throughout the Intervention Mapping process, and how much weighting should be given to their perspectives in comparison with evidence, theory and researcher judgements.

Using Intervention Mapping ensured the components are clearly based on theory and evidence overcomes criticisms regarding inadequate theory use in interventions for carers of stroke survivors [45]. Intervention Mapping uses a clearly defined and structured process to guide adequate theory and application, enhancing the likelihood of an effective intervention [31], and explicit links are made between intervention change objectives, determinants, theoretical methods, and intervention components, which are used to articulate the causal assumptions underpinning an intervention [46]. More can be learned about interventions developed using Intervention Mapping, beyond just a measure of effectiveness and interventions can be refined where necessary following evaluations.

In addition to the strengths highlighted in this section, there are some limitations. As documented by others, Intervention Mapping is clearly a time-consuming process. A particular challenge in this study was incorporating an appropriate amount of theory and evidence into the process within a specified time-period. Intervention Mapping is often presented as an unproblematic process, guiding researchers from a problem towards a solution, with reference to matrices, a stakeholder group and occasional recourse to the literature. The way the process is described suggests that there is always a volume of relevant literature, including relevant theories or that it is feasible to conduct new empirical research at numerous times throughout the process [29]. Where the development does not concern a specific behaviour in well-researched areas such as physical activity or smoking, this may not be feasible within time limited project and funding structures. Although the process was conducted according to plan, the nature of the process and the unexpected turns means that additional time, or strategies for saving time and resources could always be beneficial. In the more recent edition of guidance suggestions are provided regarding how to save time; including conducting a rapid needs assessment and choosing a more focused question from the outset [30]. Other researchers could benefit from considering these suggestions before embarking on the process.

Selecting a theory to establish the determinants to underpin the intervention proved challenging, as evidence in the logic model did not map on to a single behaviour change theory. Furthermore, the target behaviours (gaining or providing information and support) are not heavily theorised like well-researched health behaviours. This meant drawing upon limited literature about information and support seeker and provider behaviours [36, 37]. With more time, a more comprehensive search could be conducted. However, Intervention Mapping guidance does not state how the literature should be reviewed i.e. whether this should be done systematically or whether scoping is sufficient [29, 30].

The Theoretical Domains Framework [38] was used to overcome these challenges. This is a widely-used framework for mapping the factors that influence behaviour change as part of intervention development and has been used previously in the context of Intervention Mapping [47, 48]. Using this framework meant that theory-based determinants were relevant to evidence from the needs assessment. However, including all 14 domains as determinants across 15 matrices of change led to an overwhelming amount of work for the subsequent stages of intervention development. The developed intervention components target multiple determinants and include multiple theoretical methods.

To enhance and manage the intervention development, adaptations were made to the Intervention Mapping process. For example stage three where theoretical methods and practical applications are selected based on change objectives outlined in the matrices of change. Multiple matrices of change, including determinants at different levels (e.g. individual, interpersonal, and organisational) were created for mapping required behaviour change. However, as others have argued [49], this is likely to impede linkages in how behaviours interact across levels. To acknowledge the interaction required between individuals (carers and providers) to achieve the programme goal, a flow diagram based on consolidated performance objectives from different matrices was developed. This provided a basis for generating intervention ideas and a structure for the alternative tables created to map theoretical methods and practical applications. Although this deviated from the traditional process where matrices would be considered separately, the principles for selecting the theoretical methods were maintained. These were still matched to change objectives for different determinants and practical applications were still selected with consideration of parameters for effectiveness. This adaptation to the process was also advantageous as the context of this ‘interaction’ was considered, which was influential in the emergent ideas.

This example illustrates how Intervention Mapping can be used flexibly for overcoming difficulties or managing the extensive work that is created at each stage. However, as other authors have claimed, Intervention Mapping is as much an art as a science, given that ‘best solutions’ are not always available and are highly dependent on available evidence, expertise, knowledge and instincts [50]. Following thorough engagement with Intervention Mapping, we would agree with this notion and other researchers planning to use this process would benefit from being mindful of this.

### Implications and future directions

Further research is required to examine the feasibility of delivering this complex intervention in health services in the UK, then eventually other cultures and geographical settings. This will involve working with services to explore adherence to the training programme; the barriers and enablers for implementing staff training in these settings; and the level of system change needed to integrate the intervention into existing health services. If this type of intervention is to be implemented within hospitals and then in the community once stroke survivors and carers return home, policymakers, professionals and researchers need to work together in their approaches to implementation. We need to ensure that lessons are learned about supporting carers in addition to stroke survivors, to ensure the impacts of this work are realised.

## Conclusions

This paper reports the development of a proposed programme plan for the Preparing is Caring intervention which is aimed at reducing burden in carers of stroke survivors. It represents the first attempt to systematically apply theory and evidence in the development of an intervention of this nature and has been designed to support and prepare carers before, during, and following the transition from hospital to home. On reflection, we consider Intervention Mapping to be useful. However implementing this approach following training and guidance proved challenging at various stages. We recommend that others learn from the experiences presented in this paper and the need for flexibility and creativity in the application of this approach.

## Supplementary information

**Additional file 1.** Logic model of burden. The logic model of burden presents a range of behavioural and environmental factors, and their determinants that could lead to carer burden.

**Additional file 2.** Key for Additional file 1 and Additional file 5. Key to support readers to understand Additional file 1 and Additional file 5.

**Additional file 3.** Initial interview topic guide. Interview guide used in the initial interviews in group 1 and the single interviews in group 2.

**Additional file 4.** Follow up interview topic guide. Interview guide used in the second and third interviews in group 1.

**Additional file 5.** Logic model of the prioritised problem. The logic model of the prioritised problem presents a range of behavioural and environmental factors, and their determinants that could lead to carer burden associated with needing to feel prepared before during and following the transition from hospital to home.

**Additional file 6.** Logic model of change. The logic model of change outlines the pathway of intended programme effects rather than pathways to identify causes of a problem.

## Data Availability

The datasets used and/or analysed during the current study are available from the corresponding author on reasonable request.

## Abbreviations

DPP: Discharge Preparation Programme
LoTS2Care: Longer-term support stroke survivors and their carers
LSCTC: London Stroke Carer Training Course
MRC: Medical Research Council
TDF: Theoretical Domains Framework
TIRSFSP: Timing it Right Stroke Family Support Programme

## Footnotes

1 This doctoral research was conducted using the third and most current edition of the guidance available when the research commenced in 2014 [29]. During the intervention development process, a fourth edition of the guidance was introduced by Bartholomew Elderedge et al. [30]. The research continued based on the third edition; however the authors were mindful of any new material in the recent edition which could have a substantial impact on the research and content of this manuscript.
