# Supplementary figures and images for: Supporting carers of stroke survivors to reduce carer burden: development of the Preparing is Caring intervention using Intervention Mapping
Source: BMC Public Health. 2019 Oct 29;19:1408. doi: 10.1186/s12889-019-7615-2 (PMC6819539; doi:10.1186/s12889-019-7615-2)

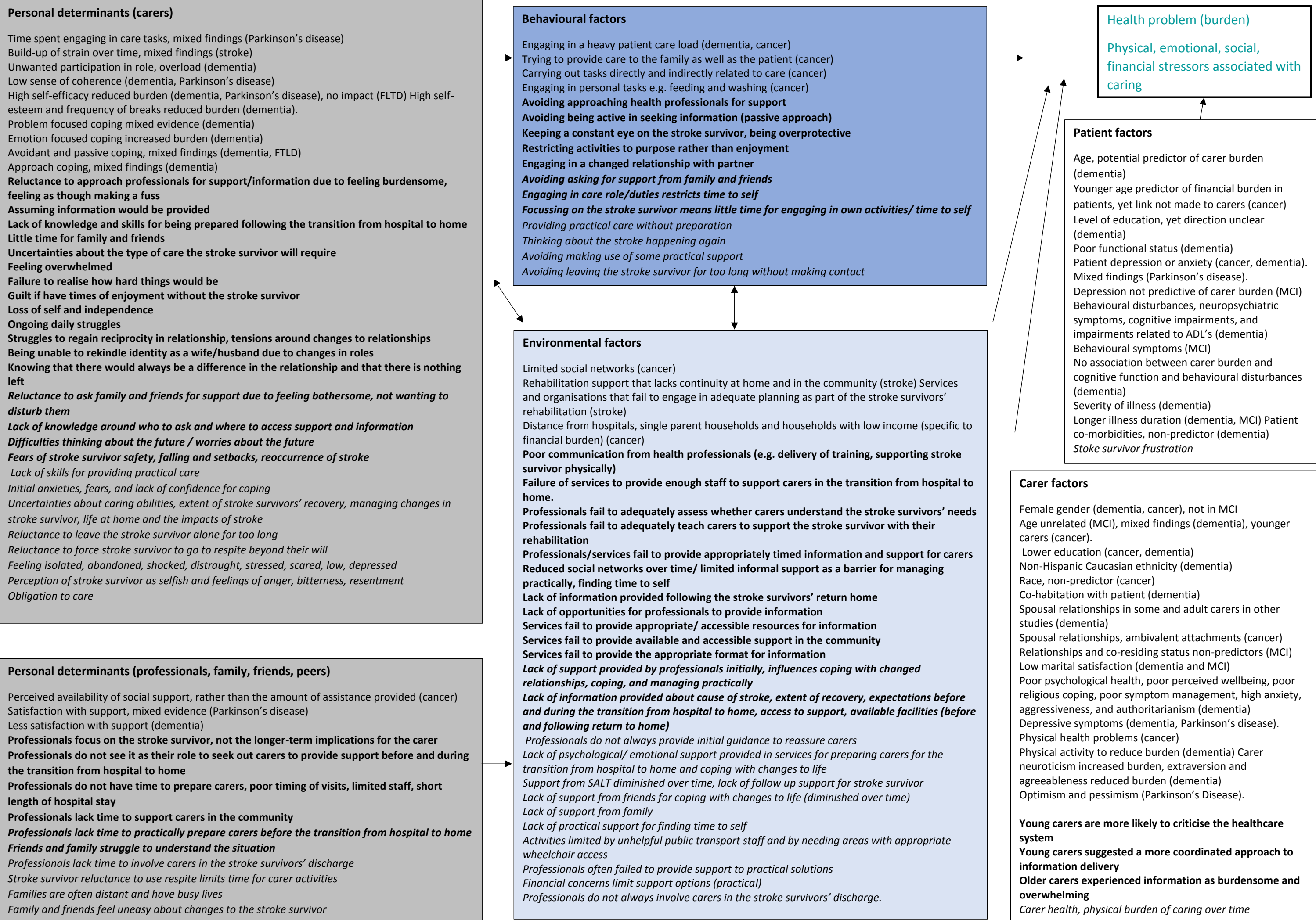

Supplement: Supplementary file 1 — Additional file 1. Logic model of burden. The logic model of burden presents a range of behavioural and environmental factors, and their determinants that could lead to carer burden. [file 12889_2019_7615_MOESM1_ESM.pdf]

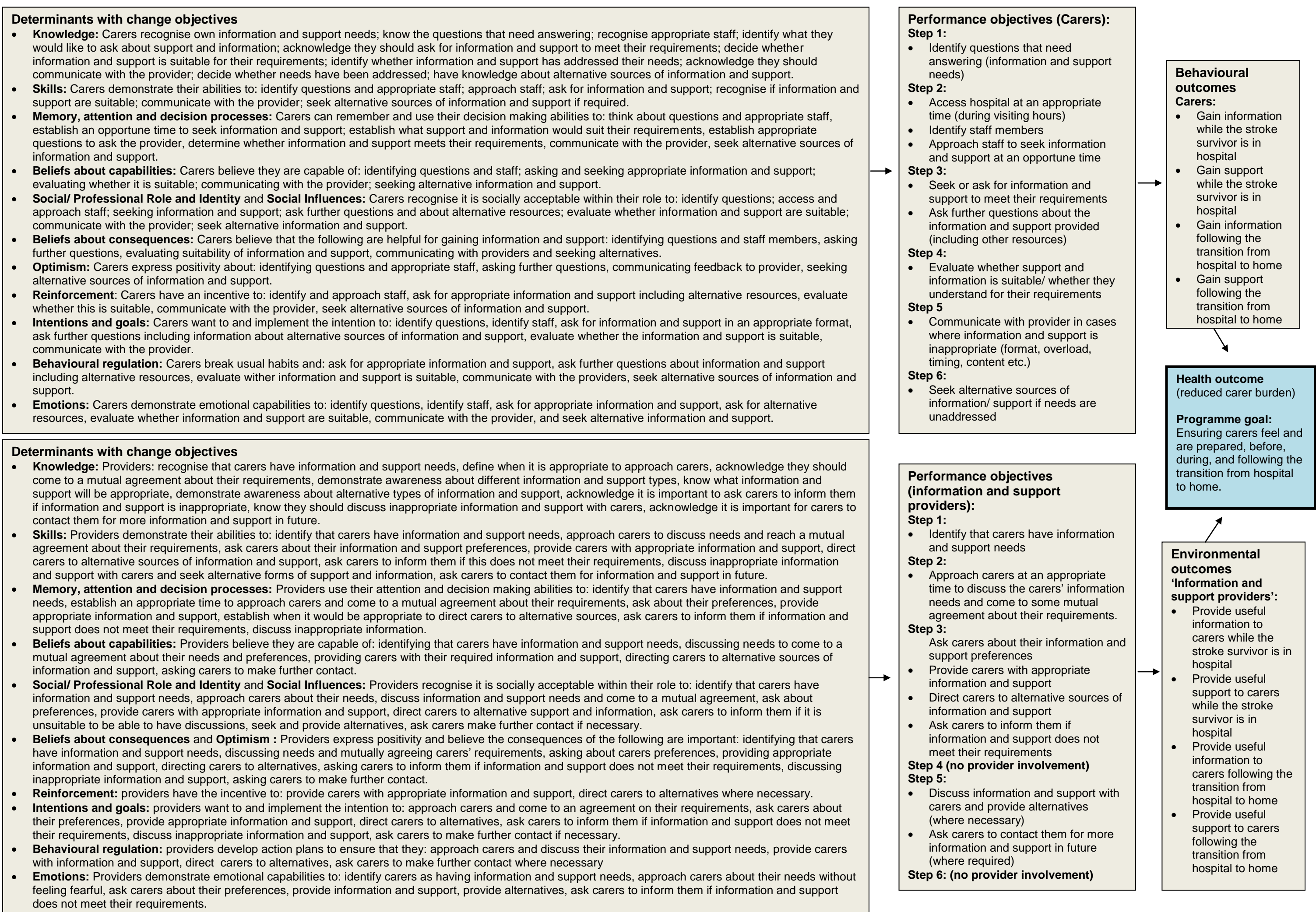

Supplement: Supplementary file 6 — Additional file 6. Logic model of change. The logic model of change outlines the pathway of intended programme effects rather than pathways to identify causes of a problem. [file 12889_2019_7615_MOESM6_ESM.pdf]
