# Supplementary material for: Supporting carers of stroke survivors to reduce carer burden: development of the Preparing is Caring intervention using Intervention Mapping
Source: BMC Public Health. 2019 Oct 29;19:1408. doi: 10.1186/s12889-019-7615-2 (PMC6819539; doi:10.1186/s12889-019-7615-2)
Supplement: Supplementary file 2 — Additional file 2. Key for Additional file 1 and Additional file 5. Key to support readers to understand Additional file 1 and Additional file 5. [file 12889_2019_7615_MOESM2_ESM.pdf]

Supplementary file 1 Key

Logic model key:

Burden review findings: Plain text with an indication of condition or conditions in a bracket
Qualitative review findings: **bold but not italic**
Empirical study findings: *italic*
Bold and *italic*- both empirical study and thematic synthesis of qualitative studies

Supplementary file 5 key

Logic model key:

Burden review findings: plain text with condition or conditions in bracket
Stakeholder meetings: text with*
Thematic synthesis of qualitative studies: **bold but not italic**
Empirical study: *italic*
Thematic synthesis of qualitative studies and empirical study: **Bold** and *italic*
