# Supplementary material for: Supporting carers of stroke survivors to reduce carer burden: development of the Preparing is Caring intervention using Intervention Mapping
Source: BMC Public Health. 2019 Oct 29;19:1408. doi: 10.1186/s12889-019-7615-2 (PMC6819539; doi:10.1186/s12889-019-7615-2)
Supplement: Supplementary file 3 — Additional file 3. Initial interview topic guide. Interview guide used in the initial interviews in group 1 and the single interviews in group 2. [file 12889_2019_7615_MOESM3_ESM.pdf]

Initial Interview Topic Guide

The study aims to:

- Gain further insight into the specific needs of carers of stroke survivors, and whether and how these change over time (e.g. type of information or support).

- Explore the health, social and emotional consequences of providing care for stroke survivors.

- Explore how carers and stroke survivors develop strategies for managing problems that they face following the stroke.

- Explore the barriers and enablers carers face in addressing their needs and the adaptive behaviours which could reduce/increase carer burden and may impact on the stroke-survivors' recovery.

- Describe in depth the social support networks of carers and stroke survivors post stroke

- Inform the Intervention Mapping process to create an evidence based intervention to support carers of stroke survivors.

In accordance with the iterative process of qualitative research, this topic guide will be refined in-line with the on-going analysis and discussion with the supervisory team. This document intends to offer guidance to the researcher conducting the interviews, rather than constraining the generation of data. Participants are free to introduce any topic(s) they wish.

The interview may cover topics that interviewees may find distressing and/or upsetting. Participants will be reminded of this prior to the interviews. It will be made clear to them that they do not have to answer any questions they do not wish to, and that they can pause or terminate the interview at any time. Due to the nature of topics to be discussed, it may be appropriate to open with general conversational

questions to help put the participant at ease with the interview situation.

The interviewer may also use aids to facilitate the interview, for instance the use of a time-line, writing down key words etc. Participants may bring items they wish to discuss in the interview, for instance calendars, appointment diaries, photos, their copies of care plans etc. The researcher will not routinely request to see such items.

Caregivers and people who have had stroke will be offered the opportunity to be interviewed separately or together.

Initial topic guide for the interview with carers of participants who have had a stroke

This topic guide is to be used in the first interview. If not all topics are covered in the first interview, they may be covered in the second interview.

General opening questions:

Could you tell me a little bit about yourself?
(Bullet points indicate possible follow-up prompts)

- Family, friends, leisure, hobbies, social activities.
- How would you describe yourself (at this time/before the stroke happened)?

Who are you caring for? (Spouse/mother/father etc.).
How long has it been since ____ had their stroke?

- Is this their first stroke?
- Have there been any other health problems alongside the stroke that require your care?

Life before your relative's stroke

Could you tell me what your life was like immediately before your relative's stroke?

- What did a typical day look like? (Employed?)
- What kinds of events/hobbies/activities did you enjoy doing?

Your relative's stroke

Could you describe to me what happened at the time of your relative's stroke?

How did your relative's stroke affect you at this early stage?

- (physically, functionally, emotionally, social consequences)?

How did you feel at this time?

Could you tell me about your relative's and your own experience in hospital? (early days post-stroke).

- Rehabilitation support given in hospital
- Information/advice/support given at this time (for both)
- What were your needs at this point? (support/information etc.).

Coming home

Could you tell me about what it was like for you and your relative coming home from hospital?

- How involved were you in the discharge process?
- How did you feel about them coming home?
- Did you feel prepared for them coming home? (why/how)
- What did you expect your life to be like? (explore new role/perceptions of caring).
- What were your needs at this point?

Could you describe a typical day when you first returned home?

- What did you think was good / difficult about coming home when your relative was first discharged? (positive and negative experiences of caring).

What support did you receive when you first came home? (both)

- Formal and informal support
- How did you find out about / access

What information / support did you find most helpful at this time?

Would you have found anything beneficial at the time that wasn't provided?

Recovery and adjustment

Could you describe how things have been since returning home?

- Process of stroke recovery over time (what has helped/hindered this process)
- Changes since discharged home (for both)
- Formal support/informal support received (both)
- Information/advice received (both)

Have your needs changed? (how/why)

Life now

Could you describe a typical day now (what do you do, how and why)

- What would make it a good/bad day – could you give me an example.
- What things do you enjoy about your daily life/what would you change?
- How is your life different now?

What activities/events do you now find meaningful/important/enjoyable?

- How have you found going out and getting about following your relative's stroke?
- Are there any activities/events that you previously found enjoyable but no longer do?

- Could you tell me about your experience of trying to resume activities/events?

Managing impacts of new role

Could you tell me how the stroke affects you now?

- Health, social, emotional consequences
- In what ways have you managed/not managed?
- Do you feel you are able to manage any challenges you face? (give an example).
- What do you need support with?
- How do you feel about ....

How have things changed over time?

- Adapted/changed the way you do everyday things? Example. What enabled you to make these changes.
- What has helped/hindered this process?

What, if any, formal support do you still receive?

- What support offered (how this has changed over time, any new engagements?)
- What do you think of this support (what is good about it/how could it be improved?)
- What was available to you/not available to you
- How did you access?

What, if any, support do you receive from family and friends? (how changed over time)

- How do you think the stroke has impacted on the rest of your /family/friends? (If appropriate).

Problem solving / resolving issues (including barriers/facilitators)

What do you feel you manage/cope with well?

Have you encountered any problems/challenges following discharge home?

- If so, could you give me an example? (Information, Financial, Employment, Time, Physical, Relationships).
- When did you first realise this was a problem/difficulty/issue?
- How did you feel about this?
- How did you react/ what did you do next?
- How have you handled/managed these?
- How did this come about? (what helped/hindered). Could you give me an example?
- Did you receive support to do this?
- How do you think you will manage this/cope with this in the future?

Unmet needs / ongoing problems/issues (including barriers/facilitators)

What do you find particularly difficult to manage/cope with?

- How have you tried to manage this / cope with this?

- How do you feel about this?

What do you feel you could do with some extra support with?
- Health, social, emotional?

What information has been most useful to you?
- what kind, what format, when, why and how

What information do you feel you need in order to manage (…specific problem highlighted)
- what kind, what format, when, why and how
- At what time point do you feel information would be useful for you?

If you experienced a problem with ..... in the future, what would you do?

Future

What are your hopes/plans for the future?
- Where do you see yourself in 6 / 12 months time?

What do you feel your needs are?

Exploring social networks

- Introduce network mapping activity
- Discuss map – could you tell me a little about each of the people/services identified. What kinds of help have they provided? Why have you placed them where you have on the diagram?
- Who was the most helpful when your friend/relative first returned home from hospital? Could you describe what they did to support you? How has this support changed over time?
- Is there anyone/any service who you expected to be more helpful for you personally?
- How did you manage to access support from …service?
- Who is particularly helpful now? Could you describe what they do to support you?
- How have family / friends / others reacted to your friend/relatives stroke? How has this changed?
- Have you grown closer to anyone since your friend/ relative's stroke? Have you grown apart from anyone since their stroke?

- Have you asked anyone for help? Did people offer to help you?
- Have you noticed any change in your relationship with each other since I last saw you?

An example of the social network mapping tool that will be used:

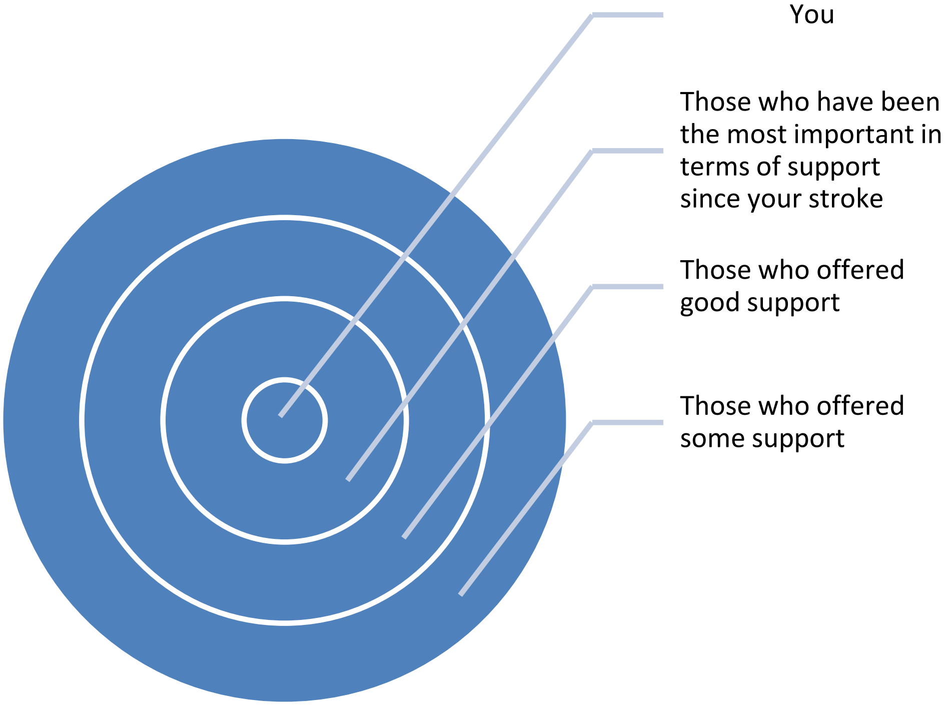

Closing questions

What advice would you give to others in similar circumstances?

Is there anything else you think I need to know about coping with life after stroke?

Do you have any questions for me?
