# Supplementary material for: Supporting carers of stroke survivors to reduce carer burden: development of the Preparing is Caring intervention using Intervention Mapping
Source: BMC Public Health. 2019 Oct 29;19:1408. doi: 10.1186/s12889-019-7615-2 (PMC6819539; doi:10.1186/s12889-019-7615-2)

Follow up interview topic guide

This follow-up interview aims to:

1. Find out more about the carers needs and whether or how these have changed over time.

2. Find out more about how carers start to come to terms with/deal with the consequences of providing care for stroke survivors.

3. Further explore how carers and stroke survivors develop strategies to overcome problems that they face within their caring role.

4. Further explore the barriers and enablers carers face in addressing their needs and the adaptive behaviours which could reduce/increase carer burden and may impact on the stroke-survivors' recovery.

5. Describe in depth the social support networks of carers and stroke survivors post stroke.

6. Inform the Intervention Mapping process to create an evidence based intervention to support carers of stroke survivors.

Each carer/ stroke survivor's journey is unique and this document intends to offer guidance to the researcher conducting the interviews rather than constraining the direction of the interview. The researcher is expected to refer to the first interviews in deciding the questions that will be most useful and relevant. In addition to the questions listed below, the second interview may also involve re-visiting some of the topics discussed with participants in the first interview. This topic guide will be refined based on emerging themes from on-going analysis.

Participants themselves are also free to introduce any topic(s) they wish.

As with the first interviews, the discussion may cover topics that interviewees find distressing and/or upsetting. Participants will be reminded of this prior to the interviews. It will be made clear to them that they do not have to answer any questions they do not wish to, and that they can pause or terminate the interview at any time.

The interviewer will also use aids to facilitate the interview, including a social network mapping tool. The social network mapping tool is adapted from the Hierarchical Mapping Technique (Antonucci, 1986). Participants will be asked to identify people

and services that they have found helpful since their stroke. These will be recorded (with the assistance of the interviewer and/or carer if required) onto the social network mapping tool (please see example of map to be used below).

General opening questions*:*

- How have you been since I last saw you?
- Has anything changed for you since we last spoke? Have things got easier/ more difficult?
- How do you feel about these changes?
- Are there things that you still struggle with? *(Explore emotional as well as practical challenges of caring)*

Exploring changes in health (physical and mental health)

- Have there been any changes in your health?
- How have you managed these changes in your daily life? Could you give me an example?
- How do you feel about these changes?
- Has anyone / anything in particular helped / hindered the changes?

Exploring changes in daily life

- Please could you describe a typical day now?
- Have you encountered any problems/challenges since we last met? Could you give me an example of how this has impacted on your daily life?
- What have you stopped doing that you did before? What has stayed the same?
- Has anyone / anything been of particular help to you as a carer? How did you access this support?
- What additional help do you still need for yourself?
- Have there been any key moments or turning points in your caring role since I last saw you?
- (If appropriate) Would you say you are getting any more / less pleasure out of life since I last saw you? In what ways?
- What do you feel your needs are?

Exploring changes in understanding / coming to terms

- (If relevant) Do you feel you have a better or a different understanding of your situation than when we met before?
- Have you had any insights or started to think differently about anything?
- How have you adapted? Are there things you feel you've learned to cope better with since we last met?
- Are there things that you used to worry about but no longer do? Are there things that still worry you?
- Do you feel you need emotional support?

Exploring social networks

- Introduce network mapping activity

- Discuss map – could you tell me a little about each of the people/services identified. What kinds of help have they provided? Why have you placed them where you have on the diagram?
- Who was the most helpful to you since we last met? Could you describe what they have done to support you? How has this support changed over time?
- Is there anyone/any service who you expected to be more helpful for you personally?
- Who is particularly helpful now? Could you describe what they do to support you?
- How have family / friends / others reacted to your friend/relatives stroke? How has this changed?
- Have you grown closer to anyone since your friend/ relative's stroke? Have you grown apart from anyone since their stroke?
- Have you asked anyone for help? Did people offer to help you?
- Have you noticed any change in your relationship with each other since I last saw you?

Closing questions

When you reflect on the last few months, is there anything you've learnt? Any advice

you’d give to others?

Do you have any questions for me?

An example of the social network mapping tool that will be used:

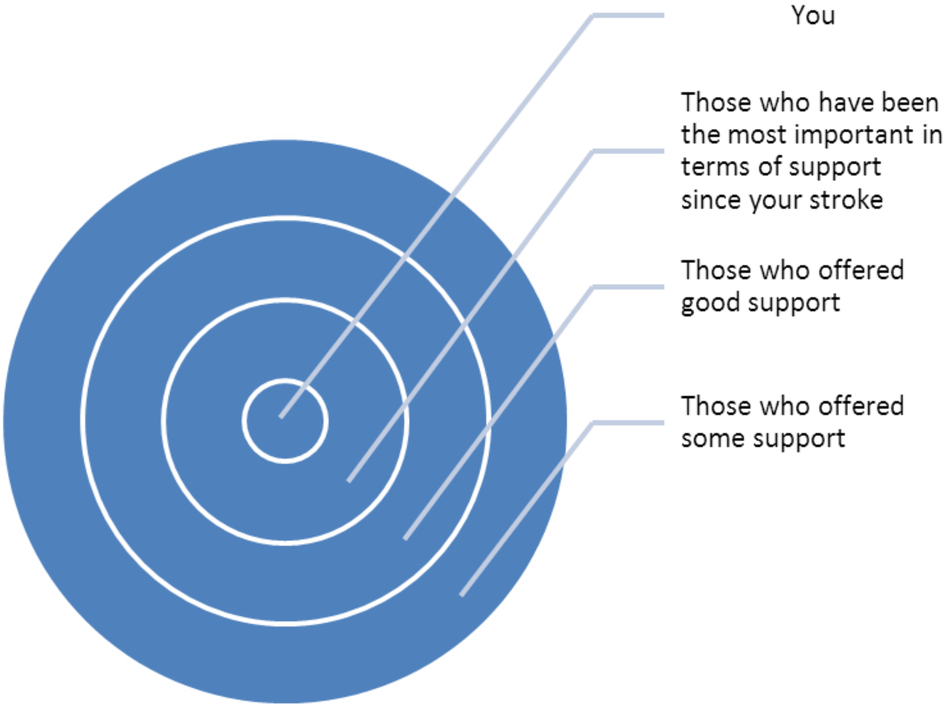

Supplement: Supplementary file 4 — Additional file 4. Follow up interview topic guide. Interview guide used in the second and third interviews in group 1. [file 12889_2019_7615_MOESM4_ESM.pdf]
