# Supplementary material for: Supporting carers of stroke survivors to reduce carer burden: development of the Preparing is Caring intervention using Intervention Mapping
Source: BMC Public Health. 2019 Oct 29;19:1408. doi: 10.1186/s12889-019-7615-2 (PMC6819539; doi:10.1186/s12889-019-7615-2)
Supplement: Supplementary file 5 — Additional file 5. Logic model of the prioritised problem. The logic model of the prioritised problem presents a range of behavioural and environmental factors, and their determinants that could lead to carer burden associated with needing to feel prepared before during and following the transition from hospital to home. [file 12889_2019_7615_MOESM5_ESM.pdf]

Personal determinants (carers)

- Carers do not want to know some things, e.g. if their partner had epilepsy*
- Carers are not always in the right frame of mind to take on some information whilst the stroke survivor is in hospital*
- Carers are too busy to read written information whilst the stroke survivor is in hospital*
- Carers are too proud to ask for information*
- **Carers do not want to bother professionals for information and support ***
- **Carers assume that information and support will be provided and therefore take a passive approach to gaining this***
- **Carers lack knowledge and skills for being prepared following the transition from hospital to home/ providing practical care**
- ***Carers lack knowledge about who and what to ask regarding support and information****
- **Carers are uncertain about the type of support required and their support needs**
- ***Carers are reluctant to ask family and friends for support due to feeling bothersome, not wanting to disturb them***
- Carers might see it as a weakness to ask for help from professionals, family or friends*
- Carers lack confidence for attending stroke groups on their own initially*
- Carers perceive that going to a group means that they are a failure*
- *Initial anxieties, fears and lack of confidence for coping*
- *Uncertainties about caring abilities, extent of stroke survivors' recovery, managing changes in stroke survivor, life at home and impacts of stroke*
- *Feeling isolated, abandoned, shocked, distraught, stressed, scared, low, depressed*
- Carers have poor or a lack of coping strategies (Stroke, Cancer, Dementia, FTLD- type of dementia) *
- **Carers lack realisation about the changes to their lives, relationships and the extent of the stroke survivors' impairments***
- **Carers lack realisation about how hard things could be***

Personal determinants (others e.g. professionals, services, friends)

- Health professionals are uncertain at stage during the stroke survivors' stay in hospital, therefore providing expectations is difficult*
- Health professionals are too busy to give carers advice in hospital *
- Professionals expect carers to cope if they have a medical background*
- Professionals focus on the physical aspects of stroke, emotion comes second*
- **Professionals focus on the stroke survivor rather than the longer term implications for the carer- wards are often therapy led***
- Professionals may lack the skills to provide support and information to carers if they have been trained medically (too much use of medical language) *
- Health professionals find it difficult to provide reassurance knowing there are poor facilities for carers*
- **Health professionals do not have time to prepare carers, poor timing of visits, limited staff, and short length of hospital stay.**
- *Professionals lack time to involve carers in the stroke survivors' discharge*
- **Health professionals do not see it as their role to seek out carers to provide support once they return home**
- **Professionals lack time to follow up carers when they return home/ provide support in the community***
- Services do not always take a family approach to providing care*
- Services have difficulties keeping staff e.g. volunteer to provide support*
- Services have difficulties getting information to carers quickly*
- Services face financial and resource constraints*
- Friends do not know how to handle the situation and often end up doing nothing*
- *Family and friends feel uneasy about changes to the stroke survivor*
- ***Friends and family struggle to understand the situation***

Behavioural factors

- **Avoiding approaching health professionals for support**
- **Avoiding being active in seeking information (passive approach)**
- Carers do not always make use of information during the stroke survivors' time in hospital *
- Carers do not seek information and support from professionals during the stroke survivors time in hospital *
- ***Avoiding asking for support from family and friends****
- Carers do not attend stroke groups*
- *Carers provide care without preparation (emotional and practical)*
- Carers do not prepare emotionally for the changes to life and relationships etc.*

Environmental factors

- Professionals in hospital do not inform the carers that the stroke survivor may change after their stroke*
- **Poor communication from professionals (e.g. delivery of training, supporting the stroke survivor physically).**
- **Professionals fail to adequately assess whether carers understand the stroke survivors' needs**
- **Professionals fail to adequately teach carers to support the stroke survivor with their rehabilitation**
- ***Professionals do not inform carers about expectations for life at home, available facilities, access to support, cause of stroke and extent of recovery****
- ***Professionals in hospital provide carers with too much information****
- ***Professionals do not provide carers with enough support that is carers specific when the stroke survivor is in hospital (emotional or practical)****
- *Professionals do not provide carers with reassurance about their abilities to care**
- **Professionals do not always include carers in the discharge process***
- **Professionals do not always provide appropriately timed information and support for carers/experience lack of opportunities to provide information**
- **Professionals do not provide carers with enough information when they return home, e.g. benefits, the stroke, groups or services***
- ***Professionals do not provide carers with enough support about how they are getting on once they return home* (influences coping with changed relationships, managing practically).***
- GPs do not provide consistent support once the stroke survivor returns home*
- **Inadequate assessments of carer needs for supporting survivor as part of service provision**
- Services fail to provide adequate home care support once the stroke survivor returns home (Stroke)
- Services failing to provide continuity of rehabilitation in the community and at home (Stroke)
- Services fail to provide psychological support once the stroke survivor leaves hospital- coping general and specific aspects e.g. relationship
- **Services failing to provide appropriate/ accessible resources for information once the stroke survivor returns home***
- **Services failing to provide appropriately formatted and timely information**
- **Services fail to provide available and accessible support in the community**
- ***Friends and family do not provide emotional or practical support (Stroke, Cancer)****

Health problem (burden)

Being/ feeling unprepared before and during the transition from hospital to home

Patient factors

- Changes in behaviours of person with condition – (Dementia, FTLD, MCI, Stroke)- crying, laughing uncontrollably, loss of inhibitions*
- Stroke survivors that lack empathy towards carer*
- Severity of illness (Dementia, MCI, Stroke*)
- Loss of speech*
- *Level of stroke survivor impairment/ extent of improvements over time (Stroke)*

Carer factors

- Young age (Cancer, Dementia, Stroke*), older age (Dementia)
- Gender- mixed findings (Dementia, Cancer, Stroke*)
- Low income (Cancer, Stroke*)
- Poor psychological health e.g. depression (Parkinson's Disease, Dementia, Stroke*)

Other factors

- *Shock, distress when the stroke initially happened**
- Not perceiving themselves as a carer initially and even for continued time following return home*
- *Disruption to their imagined future and relationships**
- Uncertainty around things getting better*
- **Uncertainty around some practical aspects***
- *Difficulties with emotional impacts attached to caring- low and depressed**
- *Entering a role with little choice (obligation to care)*
